# Effects of Different Nursing Methods on the Behavioral Response of Adult Captive Giant Pandas (Ailuropoda Melanoleuca)

**DOI:** 10.3390/ani11030626

**Published:** 2021-02-26

**Authors:** Ming-Yue Zhang, Xiao-Hui Zhang, James Ayala, Rong Hou

**Affiliations:** 1 Chengdu Research Base of Giant Panda Breeding, Chengdu 610081, China; xiaohuizhang1986@163.com (X.-H.Z.); ayalajames@msn.com (J.A.); 2 Sichuan Key Laboratory of Conservation Biology for Endangered Wildlife, Chengdu 610081, China; 3 Sichuan Academy of Giant Panda, Chengdu 610081, China

**Keywords:** nursing method, adult captive giant pandas, maternal behavior, stereotypic behavior, animal welfare

## Abstract

**Simple Summary:**

Throughout the life history of giant pandas, we have found that captive and wild giant pandas have significantly different parenting experiences. To summarize the breeding history of the Chengdu Research Base of Giant Panda Breeding over the past 30 years, we found that the parenting experience is related to the natural mating ability of captive male giant pandas. However, there are few studies on the effects of different nursing methods on the behavioral expression of captive giant pandas, mainly focusing on the behavioral development of captive giant panda cubs, but the effects on the behavioral adaptive expression and stress of adult females have not been reported. From the perspective of adult female giant pandas in the nursery period, this experiment attempts to clarify whether different nursing methods in the cub-rearing period will affect the behavioral adaptive expression of the dams. We aim to understand whether confinement mode may cause stress problems in adult captive giant pandas, and provide a scientific basis for improving the design parameters of nursery pens and improving the welfare level of captive giant pandas. Based on the current results, we find that, in the current captive environment, singleton and parent rearing are kinds of nursing methods that are in accordance with the characteristics of the natural nursing of giant pandas and have little effect on the mother–cub relationship and welfare of captive adult female giant pandas.

**Abstracts:**

Nursing protocols in giant panda conservation breeding programs often strongly deviate from the natural cub-rearing behaviors observed in situ, potentially affecting the expression of species-typical behavior in both dams and cubs. To evaluate the effects of different nursing methods on the behavioral expression of captive adult female giant pandas, it is necessary to understand such effects due to unnatural human interference to improve the welfare of giant pandas in captive breeding conditions. In this study, we selected nine captive adult female giant pandas managed with different nursing methods as the research objects. Behavioral observations were performed during the early nursing period (1–90 d). Compared with the artificially assisted nursing method, captive adult female giant pandas who adopted the singleton and parent nursing method exhibited a significantly longer time engaged in mother–cub interaction behavior and invested a significantly smaller portion of their daily time budget on pacing and railing-directed behavior in the three months postpartum. However, no significant differences in the percentage of time exhibiting maternal behaviors were noted between the different nursing methods. In conclusion, in the current captive environment, singleton and parent rearing was a kind of nursing method that matched natural cub-rearing characteristics and was less stressful for captive adult giant pandas.

## 1. Introduction

Although the ex situ conservation breeding program has basically created self-sustaining populations and genetic diversity in captive giant pandas (*Ailuropoda melanoleuca*) [1], from the perspective of welfare, confinement has certain disadvantages compared to the wild environment: (1) the living environment is fixed, single and the living space is small; (2) maternal deprivation during the nursing period is too frequent; (3) the feeding time is fixed; and (4) fresh stimulation is lacking, etc. These patterns can lead to the deprivation of essential behaviors (mate selection behavior), the suppression of instinctive behaviors (natural mating behavior), and an increase in abnormal behaviors (stereotypic pacing behavior) in giant pandas [2], and can affect the full expression of giant pandas’ normal behaviors (reproductive behavior). At present, some conservation breeding problems (such as poor natural reproductive capacity) shown by captive giant pandas have not been resolved, which has seriously affected the implementation of the wild release plan of captive giant pandas. It also does not conform to the national strategy of scientific breeding and healthy breeding of giant pandas for ex situ conservation [3,4]. The reasons for these problems are most likely to be related to the inability of the captive living environment to meet giant pandas’ various physiological needs (including the need for mate choice and environmental comfort), resulting in the inability to fully express their natural instincts, such as mate selection and mating [5,6,7].

The nursing period is a critical period for animal body development, including neural and musculoskeletal development [8]. Therefore, the lack of sufficient fresh stimulation could affect neurodevelopment [9]. Our results find that the Chengdu Research Base of Giant Panda Breeding has similar experiences in breeding male giant pandas with natural mating abilities. The four male giant pandas that could naturally mate were singletons at birth, and they were subjected to a singleton and parent nursing method throughout the nursing period. At the same time, 90% of the male giant pandas rescued in the wild could naturally mate, and most of them had experienced at least half a year’s cub-rearing with their mothers in the wild. This was related to the cub nursing strategy of wild giant pandas, because the relationship between wild female giant pandas and their cubs were very close during the nursing period (giant panda cubs were very weak at birth and had the lowest mean litter size of all ursids) [10]. In the first three months of the nursing period, the mother almost never leaves her cubs, presumably in order to give the cubs adequate care, which is conducive to their survival and later behavioral development [11]. Therefore, based on many years of breeding experience, we speculated that the mother–cub relationship was important for the physical development and behavioral expression of juvenile giant pandas [12]. The monotony of the captive artificial cub-rearing environment, and interference from various unnatural stressors (noise from tourists, replacement of enclosures, and unnatural multiple odors, etc.) could put pressure on adult female giant pandas during the nursing period [2,3,13], leading to dams failing to provide more learning and exercise opportunities for their cubs. At the same time, the expression and development of instinctual behavior in juvenile male giant pandas may also be restricted to a certain extent, and the final result might affect the expression of reproductive behavior in adulthood.

Therefore, in this study, we investigated the effects of different nursing methods on the welfare of captive adult female giant pandas. Behavioral expression and changes in the physiological responses of mothers were observed at different nursing time intervals, in order to understand captive adult female giant pandas’ characteristics of maternal behavior and the expression of abnormal behaviors in the nursing period. We evaluated the degree of adverse effects of the captive breeding mode on the giant pandas’ behavioral expression and provided a scientific basis for improving the design parameters of the nursing enclosure and the welfare of captive giant pandas.

## 2. Materials and Methods

### 2.1. Animals

We selected nine adult captive female giant pandas that successfully reproduced as the research subjects. The animals were divided into three groups: the singleton natural nursing group mothers gave birth to single cubs, and the method of cub-rearing was the parent nursing method; the singleton assisted nursing group mothers gave birth to single cubs, and the method of cub-rearing was to adopt the conventional artificially assisted method; and the twins assisted nursing group mothers produced twin cubs. For this group, the method of cub-rearing was to adopt the conventional artificially assisted method; see Table 1 for specific groups.

### 2.2. Feeding and Management of Nursery Period

Each dam and her cub used a separate nursing enclosure. The dams and their cubs lived together in the nursing enclosures until the cubs were one and a half years old. The adult female giant pandas in the nursing period lived in a set of two (5.8 m × 2.3 m) adjacent nursing enclosures. In order to keep mothers and cubs in a clean environment, there were sliding doors between the two nursing enclosures so that the dam could be moved from one enclosure to another for the enclosure to be cleaned and food provided. One side of the nursing enclosure was a wall, and other three sides were iron fences, so the shed could be cleaned from three directions. The railings of the nursing enclosure had wide spacing, which was convenient for feeders to pick up and replace the cubs. The twin mothers only nursed one cub at a time, and the two cubs were constantly swapped for feeding.

The cubs that were assigned to the singleton and natural nursing method were always kept with their mother during the nursing period, the mother and their cubs were separated less often, and the separation time was short (total time the cubs were removed for feeding was 180 min, average time away from the mother was 3.5 ± 0.5 min; total number of times the cub was removed for a health check was three times). The cubs that were subjected to the artificially assisted method were mainly raised in incubators during the nursing period and the mother animal and their cubs were often separated; however, the cubs that were subjected to the singleton assisted nursing method were almost inseparable from their mother during the first 35 days of the nursing period (total time the singleton cubs were removed for feeding was 260 min, average time away from the mother was 45.5 ± 20.5 min; total time the twin cubs were removed for feeding was 420 min, average time away from the mother was 32.8 ± 16.4 min; total number of times both the singleton and twin cubs were removed for health checks was three times). The nursing enclosure was equipped with an infrared temperature sensing camera and a normal visible light camera to ensure that we could monitor the behavior and safety of the cubs and mothers in the nursing period for 24 h (in case of accidents, where manual intervention was required).

### 2.3. Behavioral Observations and Records

We performed behavioral observations on all adult female giant pandas from cub birth to 3 months of age. We continuously observed the video from 6:00 a.m. to 6:00 p.m. on the 3rd, 12th, and 20th days, the 1st month (29th, 30th and 31st days), the 40th day, the 2nd month (59th, 60th, and 61st day), the 70th and 80th day, and the 3rd month (89th and 90th days) after the delivery of the adult female giant pandas from June 2019 to August 2020, avoiding the time that the breeder cleaned up the manure and fed the panda with bamboo, which occurred from 7:00 a.m. to 8:30 a.m. and 2:00 p.m. to 2:30 p.m. On each behavioral observation day, each panda was continuously recorded for a total of 6 h of daily activity, 2 h in the morning and 4 h in the afternoon. The key observation times without human interference were from 9:00 a.m. to 1:00 p.m. and from 3:00 p.m. to 5:00 p.m. The experimental site was recorded by a digital video system (HDR-CX680, SONY, Tokyo, Japan) for data acquisition.

The video data were analyzed using the focal animal sampling method with an instantaneous scan interval of one minute (IS). Scan samples were used to estimate the percentage of time the focal panda performed various state behaviors (feeding, resting, standing, excretion, locomotion and maternal behaviors). All-occurrence focal sampling (AO) was used to record the frequencies of different social behavioral events (stereotypic pacing and railing-directed behaviors). State behavioral data were converted to a percentage of observed time, and event behaviors were calculated as the actual number of occurrences of the behavior (for specific behaviors and definitions see Table 2).

**Table 2:** Behavioral categories and their definitions.

Overarching Behavioral Category	Behavioral Categories	Definitions
Mother–cub behaviors	Resting (%) [2]	Individuals remain stationary in various postures, lying, or sitting without changing position, which is called the non-active state. Includes holding the cub and resting while the cub is on the ground.
	Feeding (%) [2]	Consumption, or processing for consumption, of provisioned food, including bamboo and supplementary diet items in a variety of postures while holding the cub, including lateral lying, ventral lying, lying and sitting.
	Locomotion (%) [2]	Any kind of directional travel between points, including walking, standing, running, grooming, climbing, changing position, nursing, dropping cubs and any other form of interaction between the mother and the infant.
	Maternal behaviors (%) [14]	Includes grooming the cub; nursing the cub; licking the cub’s anogenitals; holding the cub. Mother provides physical comfort to infant as a response to its vocalization or movement.
	Mother–cub interaction behavior (%)	All the state behaviors of mother-infant. Includes resting, feeding and locomotion.
Behavior of mother during separation from her cubs	Stereotypic behaviors (times/min) [2]	Animal moves back and forth or walks in circles in a repetitive way at least three times, including pacing (continuous walking back and forth following the same path); circling (walking following a defined route by placing feet in the same position each time); railing-directed movements (animal waits at the door restlessly, standing on their hind legs and putting their fore legs on the railing, or scratching the railing to indicate the expectation of her cub being delivered). These actions are repeated at least 3 times in succession.
	Stand and Locomotion (%) [2]	Individuals in various non-stationary states, any kind of directional travel between points, including walking, running and climbing without placing their feet in the same position each time and following the same path. Stand and Locomotion is called the active state.
	Resting (%) [2]	Individuals remain stationary in various postures, lying, sitting or standing without changing position, which is called the non-active state.
	Feeding and Drinking (%) [2]	Consumption, or processing for consumption, of provisioned food, including bamboo and supplementary diet items and drinking water in a variety of postures, including lateral lying, ventral lying, lying and sitting.
	Excretion (%) [2]	Urinating and defecating while in a squat, leg cock, handstand, or standing posture on the wall or ground.

### 2.4. Statistical Analysis

The data collected from the behavioral tests were analyzed with the Statistic Package for the Social Sciences (SPSS 23.0; software IBM Institute Inc., Chicago, IL, USA). Because the sample size was small, data were analyzed for agreement with a normal distribution using the Shapiro–Wilk test, and we used the Explore function in Descriptive Statistics to test the homogeneity of the variance in the data.

Through analysis, we found that all behavioral data met the conditions of a normal distribution and homogeneity of variance. Therefore, we performed a univariate process under a General Linear Model (GLM) for maternal behavioral data using the within-subjects factor nursing methods (singleton natural nursing vs. singleton assisted nursing vs. singleton assisted nursing) and the between-subjects factor nursing time (1st month vs. 2nd month vs. 3rd month after parturition). The fixed effects included nursing time, nursing method, and their interaction (nursing time × nursing method). To account for multiple comparisons, we used Duncan’s post hoc new multiple range tests, which were conducted in case of significant main effects.

Since the sample size of each group was only three, we could not use GLM to analyze the data, so we used traditional non-parametric tests or the compare means method to perform pairwise comparison. Through analysis, we found that all behavioral data meet the conditions of normal distribution and homogeneity of variance, so we used the compare means method to compare the percentages of time engaged in different state behaviors (resting behavior and feeding behaviors) and the frequency of stereotypic behaviors displayed by the different experimental groups of female pandas (singleton natural nursing group, singleton assisted nursing group and twins assisted nursing group) in the first three months (1-90 d) of the cub-rearing periods. The differences between the two nursing methods (singleton natural nursing vs. singleton assisted nursing and singleton natural nursing vs. twins assisted nursing) in different nursing stages (3rd, 12th, 20th, 30th, 40th, 60th and 70th, 80th and 90th day after parturition) were evaluated using two-tailed Independent Sample T tests. Then, we used one-way analysis of variance (ANOVA) to examine the effects of the nursing time on the behavioral changes and different behaviors of different experimental groups of female pandas. Multiple comparisons were performed using the least-significant difference (LSD) method. Differences between groups were considered to be statistically significant if the associated *p*-value did not exceed 0.05. The values in the text are expressed as the mean ± standard deviation (S.D.).

## 3. Results

**Figure 1:**
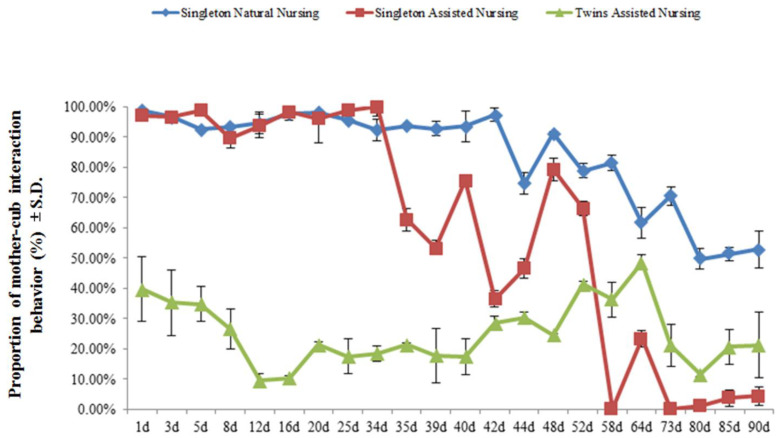
The percentage of time engaged in mother–cub interaction behavior during the early stage of the nursing period. Data were presented as mean ± S.D. (standard deviation). Mother–cub period: mother and cub together; mother-only period: the cub was removed and the mother was separated from her cub. Percentage of time engaged in mother–cub interaction behavior = time engaged in mother–cub period/(time engaged in mother–cub period ± time engaged in mother-only period) × 100%.

Figure 1 demonstrates that the different nursing modes resulted in significant differences in the average time spent between the dam and her cub. Among them, the average time females who adopted the singleton and natural nursing method were engaged in mother–cub interaction behavior (84.12 ± 16.55%) was significantly greater than that of females who adopted the artificial assisted nursing method (singleton: 60.13 ± 38.93%; twins: 25.24 ± 10.54%) (*t* = 2.660, df = 42, *p* < 0.01/*t* = 14.075; df = 42, *p* < 0.01) (Figure 1). As the cubs developed, the percentage of time engaged in mother–cub interaction was significantly reduced, regardless of the nursing method (singleton natural nursing group: 98.78 ± 0.22% vs. 52.88 ± 12.35%, *t* = 6.812, df = 2, *p* < 0.05; singleton assisted nursing group: 97.15 ± 1.45% vs. 4.45% ± 2.40%, *t* = 41.649, df = 2, *p* < 0.01; twins assisted nursing group: 39.90 ± 10.20% vs. 21.33 ± 8.55%, *t* = 19.493, df = 2, *p* < 0.01) (Figure 1). 

The interaction between different nursing methods in the group and the different nursing times between the groups had no effect on maternal behaviors (F(4.8) = 0.151, *p* = 0.962).

**Figure 2:**
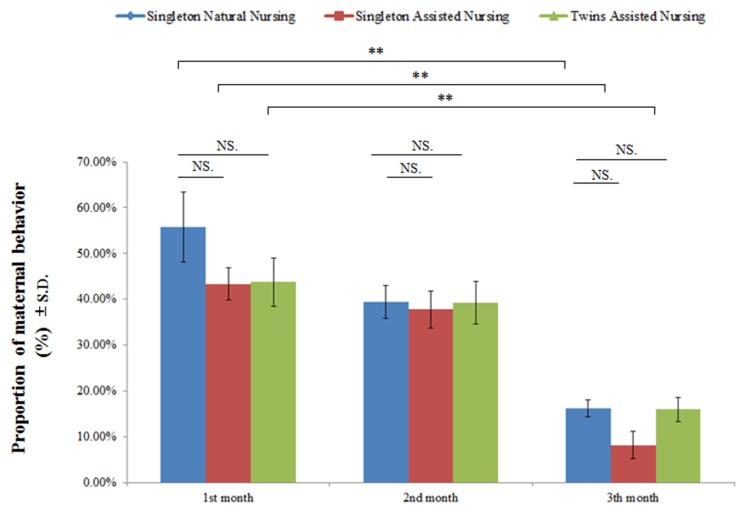
Comparison of the maternal behaviors of different experimental group pandas in the mother–cub period. Data were presented as the mean ± S.D. (standard deviation). NS (no significant difference). *p* > 0.05; ** *p* < 0.01. *n* = 8 per group.

Figure 2 and Table 3 demonstrate that, regardless of the nursing method, there was no significant difference in the performance of maternal behavior by adult female giant pandas in captivity (F(2.8) = 1.024, *p* = 0.365); however, nursing time can significantly affect the maternal behavioral expression (F(2.8) = 48.582, *p* < 0.01) (Figure 2 and Table 3). As the cubs developed, the time spent by the dams in caring for their cubs declined significantly (singleton natural nursing group: F(2.23) = 15.722, *p* < 0.01; singleton assisted nursing group: F(2.23) = 20.968, *p* <0.01; twins assisted nursing group: F(2.23) = 13.316, *p* < 0.01) (Figure 2 and Table 3).

Percentage of time engaged in maternal behaviors = time engaged in maternal behaviors by mothers/time engaged in mother–cub period × 100%.

**Figure 3:**
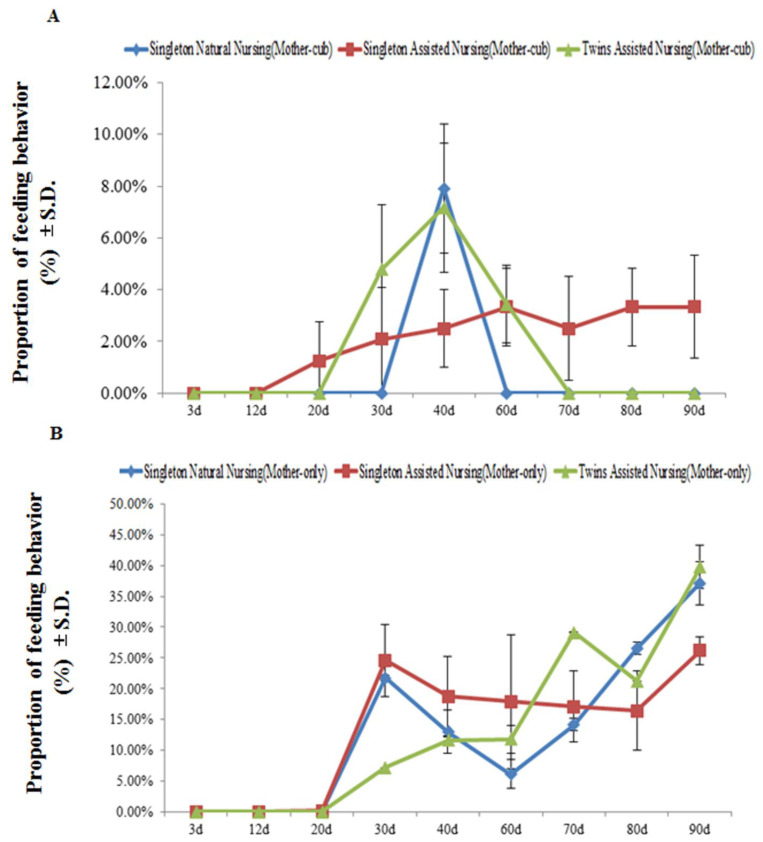
The percentage of time engaged in mother feeding behavior during the mother–cub (panel **A**) period and mother-only period (panel **B**). Data were presented as the mean ± S.D. (standard deviation). *n* = 3 per group.

Figure 3 demonstrates that, at least during the observation period, in the stage when the mother animals and their cubs were together, no feeding behavior was observed in the giant pandas in all experimental groups within the first 12 days after parturition. As cubs developed, the captive adult female giant pandas that adopted the artificially assisted singleton nursing method began to show feeding behaviors from the 20th day after parturition (singleton assisted nursing group: 1.21 ± 0.79%; twins assisted nursing group: 4.78 ± 0.32%) (Figure 3A), whereas pandas that adopted the natural nursing method began to show feeding behaviors on the 40th day after parturition (singleton natural nursing group: 7.88 ± 0.45%) (Figure 3A). In addition, no feeding behavior was observed in the singleton natural nursing group and twins assisted nursing group until the 60th day and the 70th day after parturition, respectively, but this behavior was always observed in the singleton assisted nursing group (Figure 3A). When the cubs were removed and the mothers were separated from their cubs, none of the experimental groups showed feeding behavior until the 20th day after parturition. As cubs developed, the time of the feeding behaviors of all the experimental group pandas increased significantly from the 3rd to the 90th day (singleton natural nursing group: F(8.26) = 71.178, *p* < 0.01; singleton assisted nursing group: 3rd vs. 90th day: F(8.26) = 138.566, *p* < 0.01; twins assisted nursing group: F(8.26) = 329.163, *p* < 0.01) (Figure 3B).

Percentage of time engaged in mother feeding behaviors during the mother–cub period = time engaged in mother feeding behaviors during the mother–cub period/time engaged in the mother–cub period × 100; percentage of time engaged in mother feeding behaviors when mother and cub were together = time engaged in mother feeding behaviors when the cub was removed and the mothers were separated from their cub/time engaged in the mother-only period × 100.

**Figure 4:**
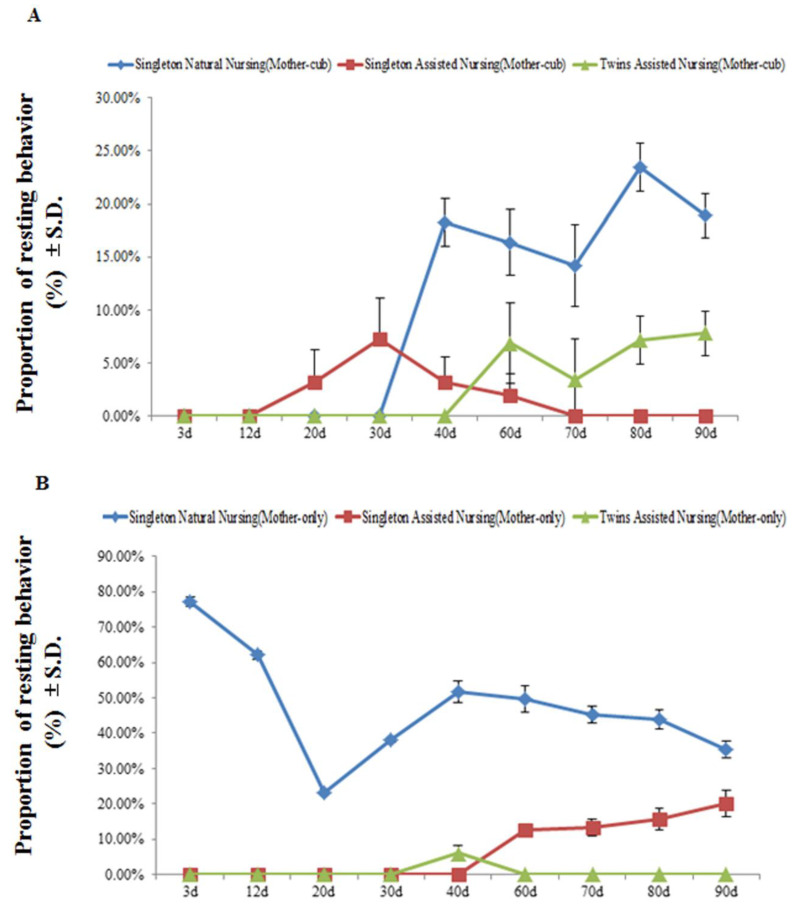
The percentage of time the mother engaged in resting behavior during the mother–cub (panel **A**) period and mother-only period (panel **B**). Data were presented as the mean ± S.D. (standard deviation). *n* = 3 per group.

Figure 4 and Table 4 demonstrate that, at least during the observation period, at the stage when the mother animals and their cubs were together, no resting behavior was observed in the giant pandas in all experimental groups within the first 12 days after parturition. As cubs developed, the resting behavior time in the singleton natural nursing group and twins assisted nursing group pandas increased significantly from the 3rd to the 90th day after parturition (singleton natural nursing group: F(8.26) = 123.338, *p* < 0.01; twins assisted nursing group: F(8.26) = 44.009, *p* < 0.01) (Figure 4A). Among the groups, from the 40th to the 80th day of the nursing period, the resting behavior time of the giant pandas in the singleton natural nursing group was significantly greater than that in the singleton and twins assisted nursing group (Table 4). When the cubs were removed and the mothers were separated from their cubs, as cubs developed, the resting behavior time in the singleton natural nursing group decreased significantly from the 3rd to the 90th day after parturition (singleton natural nursing group: F(8.26) = 57.679, *p* < 0.01) (Figure 4B); however, the resting behavior time in the singleton assisted nursing group decreased significantly (singleton assisted nursing group: F(8.26) = 89.882, *p* < 0.01) (Figure 4B). In addition, the resting behavior time of the giant pandas in the singleton natural nursing group was significantly greater than that in the singleton and twins assisted nursing group during the first 90 days of the nursing period (Table 4).

**Figure 5:**
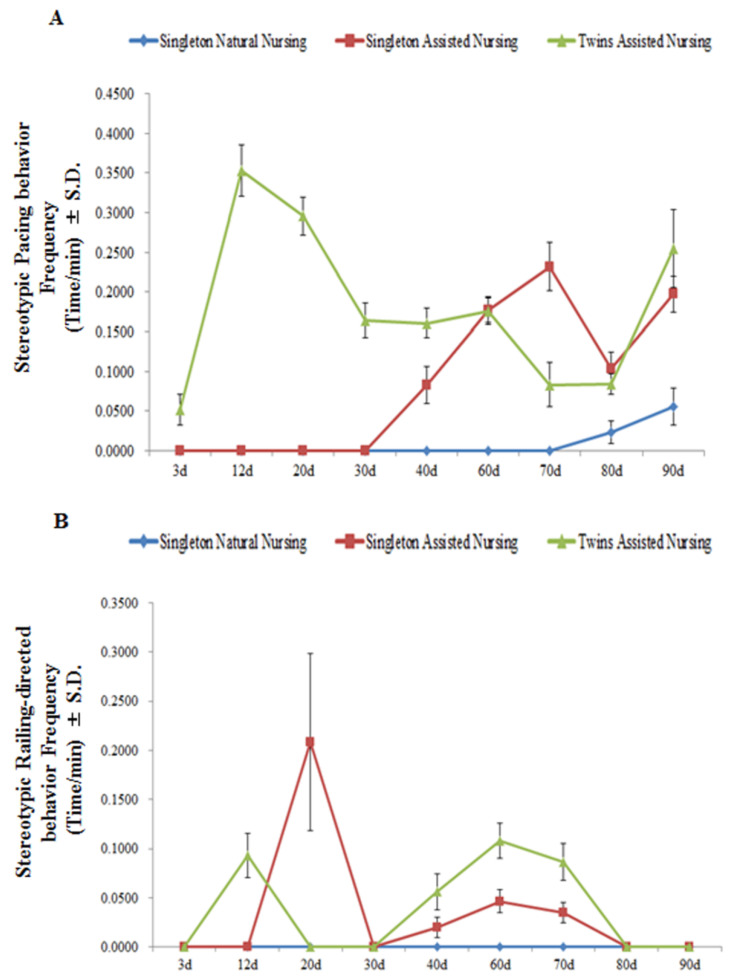
Changes in panda mothers’ pacing behaviors (panel **A**) and railing-directed behaviors (panel **B**) when the cubs were removed from the mothers. Data were presented as the mean ± S.D. (standard deviation). *n* = 3 per group.

As shown in Figure 5 and Table 5, during the first 70 days of the nursing period, removing the cubs did not lead to stereotypic behaviors by the dams in the singleton natural nursing group (pacing and railing-directed behaviors). As cubs developed, the frequencies of the pacing behaviors in the singleton assisted nursing group significantly increased from the 40th day to the 70th day, and significantly decreased on the 80th day of the nursing period (F(8.26) = 109.921, *p* < 0.01). In the twins assisted nursing group, the frequency of this behavior first significantly increased from the 12th day and then significantly decreased from the 20th to the 80th day (F(8.26) = 51.986, *p* < 0.01) (Figure 5A). However, the values were significantly higher than that of dams in the singleton natural nursing group (Table 5). The frequency of railing-directed behavior in the singleton assisted nursing group and twins assisted nursing group exhibited the same trend of first significantly increasing, then decreasing, then significantly increasing, and finally decreasing (singleton assisted nursing group: (F(8.26) = 27.302, *p* < 0.01; twins assisted nursing group: (F(8.26) = 37.046, *p* < 0.01) (Figure 5B)). The values were also significantly higher than that of dams in the singleton natural nursing group on the 20th, 40th, 60th, and 70th days after parturition (Table 5). No stereotypic behaviors were observed in the captive adult female giant pandas in the singleton natural nursing group from the 3rd to the 70th day of the nursing period (Figure 5A,B).

## 4. Discussion

In this study, we found that adopting natural nursing and hand nursing methods in the nursing period does indeed affect the behavioral expression of captive adult female giant pandas to varying degrees. At the same time, the expression of the dams’ nursing behavior was also different when using artificially assisted cub-rearing to raise single and twin cubs. This was similar to the results at the Vienna Zoo, which used natural nest breeding to raise single cubs and twins successfully. They found that the dams that raised twins had less rest time than singletons in the first month postpartum. At the same time, the percentage of time engaged in mother–cub interaction behavior with twins was higher than singletons (the rest time was lower) [15]. In this study, we found that, during the period when workers took the cubs for weighing and health checks, the dams who adopted the natural nursing method spent significantly less time pacing than the females who adopted the artificially assisted nursing method. Compared with the artificially assisted nursing method, the natural nursing method did not result in the dam exhibiting railing-directed behavior, but instead resulted in significantly increased resting behavior. This might indicate that adopting the method of natural nursing lessened the anxiety of the captive adult female giant pandas when separated from their cubs. When behavioral ecologists observed the nursing behavior of giant pandas in the wild, it was found that dams and their cubs were rarely separated from the birth of the baby to the age of three months, and the percentage of time the adult female panda spent being active was lower than that of non-parenting pandas. After the cub reaches the age of three months, the percentage of activity tends to be the same. As the cubs developed, the average time that the dam spent outside the cave and the average time they spent in an inactive state increased significantly, that is, the total time spent outside the cave increased [11,16]. The results of this study seem to indicate that the method of natural rearing during the nursing period was in line with the characteristics of wild giant pandas and caused less anxiety to the mother. In addition, judging from where the adult female captive giant pandas chose to excrete, all the experimental pandas during the nursing period selected a place of excretion far away from their cubs. This was consistent with the observation in the field that wild dams excrete away from the cavity; however, the range allowed for other activities is not be too far from the hole, which not only saves the physical strength of the mother animal, but also ensures the safety of the cubs and the cleanliness of the nursery environment [17,18].

For adult female giant pandas in captivity that use artificially assisted parenting to raise single litters, this study did not find that the rearing experience significantly affects the maternal and stereotyped behaviors of captive adult female giant pandas. Regardless of whether the panda was a primiparous mother or a multiparous mother, there was no significant difference in the time spent undertaking in maternal behavior and the frequency of stereotyped behaviors. However, it was unclear whether the nursing experience would affect the maternal behaviors and stereotypic behaviors of mothers who were subjected to two rearing methods during the nursing period. However, the results of the study by Snyder et al. (2016) show that multiparous female giant pandas spent a longer time nursing, grooming, and cuddling cubs than primiparous mothers [13]. This also showed that rearing singletons vs. twins had an impact on the behavioral expression of adult giant pandas in captivity [14]. In addition, judging from the performance of stereotyped behaviors, the nursing pattern caused the most serious damage to the welfare of the adult captive female giant pandas. This emphasizes the importance of parenting and keeping maternal deprivation to as low a level as possible to maintain the maternal behavioral expression and welfare of captive giant pandas. However, it was unclear whether the nursing experience affects the stereotypic behavior of mothers, because our previous research on the stereotypic behaviors of captive sows during pregnancy found that long-term space restriction experiences significantly increase the frequency of stereotypic behaviors in sows and lead to a decline in their welfare [19].

Animal welfare scholars believe that the emergence of stereotypes might indicate that animals are not adapting to their living environment and are suffering from psychological distress [20]. In this study, we found that the artificially assisted cub-rearing method reduced the percentage of time engaged in mother–cub interaction behavior in the three months postpartum, resulting in frequent stereotypic behaviors such as pacing and railing-directed behaviors after the mother and the cub were separated. It was thus easy to deduce from this phenomenon that the occurrence of stereotyped behavior could not be separated from the role of motivation, because most psychological representations of animals are regarded as the basis of the motivation for a behavior to occur [21]. This study proved that prolonged maternal deprivation in early childhood also increased the occurrence of stereotypic behavior in captive adult female giant pandas. Thus, maternal deprivation might produce changes in temperament or stress responsiveness that influence life-long behavioral responses to stressors, or may cause neural changes that make animals more prone to “inappropriate” behavior. In order to give the dams producing twins sufficient rest time, the percentage of time engaged in mother–cub interaction behavior was not more than 50%, although long periods of maternal deprivation led to the dams spending a longer time pacing and expressing more railing-directed behaviors [8]. This also showed that the relationship between the mother and the cubs in the early stages of the nursing period not only profoundly affects the behavioral development and expression of the cubs [12], but also affects the behavioral expression and psychological status of adult captive giant pandas.

## 5. Conclusions

In this study, we found that different nursing methods could significantly affect the mother’s behavioral response and the relationship between captive adult female giant pandas and their cubs in the three months postpartum. The artificially assisted nursing method significantly increased the time the dam spent engaged in pacing behavior and the frequency of railing-directed behavior, and increased the length of the maternal deprivation, causing the captive adult female giant pandas to develop severe psychological anxiety, damaging the relationship between the mothers and their cubs. In the current captive environment, singleton and parent rearing is a nursing method that is practiced in accordance with natural cub-rearing characteristics and is conducive to the welfare of adult captive giant pandas.

## Figures and Tables

**Table 1:** Experimental group from Chengdu Research Base of Giant Panda Breeding (CHENPANDA) (Chengdu, China).

Nursing Group	Name	Studbook	Parity	Sex of the Cubs
Singleton Natural Nursing Group	Zhao Mei	990	Primiparous	Male
Singleton Natural Nursing Group	Yuan Run	853	Primiparous	Female
Singleton Natural Nursing Group	Ai Li	811	Primiparous	Female
Singleton Assisted Nursing Group	Xing Ya	879	Primiparous	Female
Singleton Assisted Nursing Group	Ji Li	671	Multiparous	Female
Singleton Assisted Nursing Group	Zhi Zhi	763	Multiparous	Male
Twins Assisted Nursing Group	Xiao Yatou	635	Multiparous	Male; Female
Twins Assisted Nursing Group	Cheng Da	824	Multiparous	Female; Female
Twins Assisted Nursing Group	A Bao	801	Multiparous	Male; Female

Singleton Natural Nursing Group: Mother animals adopt natural nursing method; Singleton Assisted Nursing Group: Mother animals adopt artificially assisted singleton nursing method; Twins Assisted Nursing Group: Mother animals adopt artificially assisted twin nursing method.

**Table 3:** Maternal behavioral observations of the different nursing methods of groups of captive giant panda mothers were performed on the 1st, 2nd and 3rd month of the mother–cub period. The data were presented as mean ± S.D (standard deviation).

Nursing Method	1st Month	2nd Month	3rd Month
Singleton and natural nursing method	48.64 ± 7.63%	43.30 ± 3.60%	16.73 ± 1.86%
Singleton assisted nursing method	43.13 ± 3.58%	41.69 ± 4.11%	6.73 ± 1.10%
Twins assisted nursing method	48.64 ± 5.29%	41.76 ± 4.67%	14.46 ± 2.60%

**Table 4:** Comparison of resting behavioral responses of different nursing group panda mothers during the different stages of infancy.

Comparison between Different Nursing Methods	Stage of Nursing Period								
	3rd Day	12th Day	20th Day	30th Day	40th Day	60th Day	70th Day	80th Day	90th Day
G 1 vs. G 2 (%) (Mother-only period)	77.22 ± 4.14 vs. 0.00 ± 0.00	62.44 ± 3.39 vs. 0.00 ± 0.00	23.32 ± 1.94 vs. 0.00 ± 0.00	38.29 ± 2.47 vs. 0.00 ± 0.00	51.69 ± 2.42 vs. 0.00 ± 0.00	49.68 ± 2.08 vs. 12.34 ± 2.34	45.35 ± 5.66 vs. 13.08 ± 2.47	43.77 ± 4.77 vs. 15.59 ± 2.76	35.22 ± 3.41 vs. 20.11 ± 1.30
Independent Sample T tests	*t* = 32.302, *p* < 0.01	*t* = 31.866, *p* < 0.01	*t* = 20.850, *p* < 0.01	*t* = 26.868, *p* < 0.01	*t* = 36.925, *p* < 0.01	*t* = 20.644, *p* < 0.01	*t* = 9.057, *p* < 0.01	*t* = 8.860, *p* < 0.01	*t* = 7.164, *p* < 0.01
G 1 vs. G 3 (%) (Mother-only period)	77.22 ± 4.14 vs. 0.00 ± 0.00	62.44 ± 3.39 vs. 0.00 ± 0.00	23.32 ± 1.94 vs. 0.00 ± 0.00	38.29 ± 2.47 vs. 0.00 ± 0.00	51.69 ± 2.42 vs. 6.06 ± 1.80	49.68 ± 2.08 vs. 0.00 ± 0.00	45.35 ± 5.66 vs. 0.00 ± 0.00	43.77 ± 4.77 vs. 0.00 ± 0.00	35.22 ± 3.41 vs. 0.00 ± 0.00
Independent Sample T tests	*t* = 32.302, *p* < 0.01	*t* = 31.866, *p* < 0.01	*t* = 20.850, *p* < 0.01	*t* = 26.868, *p* < 0.01	*t* = 26.189, *p* < 0.01	*t* = 41.365, *p* < 0.01	*t* = 13.888, *p* < 0.01	*t* = 15.896, *p* < 0.01	*t* = 17.865, *p* < 0.01
G 1 vs. G 2 (%) (Mother–cub period)	0.00 ± 0.00 vs. 0.00 ± 0.00	0.00 ± 0.00 vs. 0.00 ± 0.00	0.00 ± 0.00 vs. 3.70 ± 0.85	0.00 ± 0.00 vs. 7.07 ± 1.69	18.36 ± 2.57 vs. 3.34 ± 1.17	16.59 ± 2.15 vs. 2.09 ± 0.50	14.23 ± 2.22 vs. 0.00 ± 0.00	23.28 ± 1.20 vs. 0.00 ± 0.00	18.74 ± 1.95 vs. 0.00 ± 0.00
Independent Sample T tests			*t* = -7.501, *p* < 0.01	*t* = -7.233, *p* < 0.05	*t* = 9.211, *p* < 0.01	*t* = 11.356, *p* < 0.01	*t* = 11.094, *p* < 0.01	*t* = 33.556, *p* < 0.01	*t* = 16.610, *p* < 0.01
G 1 vs. G 3 (%) (Mother–cub period)	0.00 ± 0.00 vs. 0.00 ± 0.00	0.00 ± 0.00 vs. 0.00 ± 0.00	0.00 ± 0.00 vs. 0.00 ± 0.00	0.00 ± 0.00 vs. 0.00 ± 0.00	18.36 ± 2.57 vs. 0.00 ± 0.00	16.59 ± 2.15 vs. 6.10 ± 2.10	14.23 ± 2.22 vs. 3.75 ± 0.96	23.28 ± 1.20 vs. 7.19 ± 0.92	18.74 ± 1.95 vs. 7.58 ± 0.98
Independent Sample T tests					*t* = 12.372, *p* < 0.01	*t* = 6.029, *p* < 0.01	*t* = 7.498, *p* < 0.01	*t* = 18.446, *p* < 0.01	*t* = 8.839, *p* < 0.01

G 1: Singleton natural nursing group; G 2: singleton assisted nursing group; G 3: twins assisted nursing group. Data were presented as mean ± S.D. (standard deviation). *n* = 3 per group.

**Table 5:** Comparison of stereotypic behavioral response of different nursing group panda mothers during the different stages of infancy.

Comparison between Different Nursing Methods	Stage of Nursing Period								
	3rd Day	12th Day	20th Day	30th Day	40th Day	60th Day	70th Day	80th Day	90th Day
G 1 vs. G 2 (time/min) (Pacing behavior)	0.0000 ± 0.0000 vs. 0.0000 ± 0.0000	0.0000 ± 0.0000 vs. 0.0000 ± 0.0000	0.0000 ± 0.0000 vs. 0.0000 ± 0.0000	0.0000±0.0000 vs. 0.0000 ± 0.0000	0.0000 ± 0.0000 vs. 0.0826 ± 0.0229	0.0000 ± 0.0000 vs. 0.1770 ± 0.0156	0.0000 ± 0.0000 vs. 0.2322 ± 0.0299	0.0234 ± 0.0136 vs. 0.1030 ± 0.0219	0.0555 ± 0.0234 vs. 0.1977 ± 0.0225
Independent Sample T tests					*t* = −6.244, *p* < 0.05	*t* = −19.590, *p* < 0.01	*t* = −13.456, *p* < 0.01	*t* = −5.346, *p* < 0.01	*t* = −7.588, *p* < 0.01
G 1 vs. G 3 (time/min) (Pacing behavior)	0.0000 ± 0.0000 vs. 0.0522 ± 0.0191	0.0000 ± 0.0000 vs. 0.3531 ± 0.0322	0.0000±0.0000 vs. 0.2956±0.0244	0.0000±0.0000 vs. 0.1646 ± 0.0219	0.0000 ± 0.0000 vs. 0.1611 ± 0.0185	0.0000 ± 0.0000 vs. 0.1766 ± 0.0178	0.0000 ± 0.0000 vs. 0.0831 ± 0.0279	0.0234 ± 0.0136 vs. 0.0840 ± 0.0129	0.0555 ± 0.0234 vs. 0.2550 ± 0.0493
Independent Sample T tests	*t* = −4.734, *p* < 0.01	*t* = −19.000, *p* < 0.01	*t* = −21.026, *p* < 0.01	*t* = −13.035, *p* < 0.01	*t* = −15.057, *p* < 0.01	*t* = −17.196, *p* < 0.01	*t* = −5.154, *p* < 0.05	*t* = −5.587, *p* < 0.01	*t* = −6.326, *p* < 0.01
G 1 vs. G 2 (time/min) (Railing-directed behavior)	0.0000 ± 0.0000 vs. 0.0000 ± 0.0000	0.0000 ± 0.0000 vs. 0.0000 ± 0.0000	0.0000 ± 0.0000 vs. 0.2084 ± 0.0902	0.0000 ± 0.0000 vs. 0.0000 ± 0.0000	0.0000 ± 0.0000 vs. 0.0198 ± 0.0100	0.0000 ± 0.0000 vs. 0.0465 ± 0.0116	0.0000 ± 0.0000 vs. 0.0346 ± 0.0104	0.0000 ± 0.0000 vs. 0.0000 ± 0.0000	0.0000 ± 0.0000 vs. 0.0000 ± 0.0000
Independent Sample T tests			*t* = −4.002, *p* = 0.057		*t* = −3.464, *p* < 0.05	*t* = −6.928, *p* < 0.01	*t* = −5.747, *p* < 0.01		
G 1 vs. G 3 (time/min) (Railing-directed behavior)	0.0000 ± 0.0000 vs. 0.0000 ± 0.0000	0.0000 ± 0.0000 vs. 0.0929 ± 0.0224	0.0000 ± 0.0000 vs. 0.0000 ± 0.0000	0.0000 ± 0.0000 vs. 0.0000 ± 0.0000	0.0000 ± 0.0000 vs. 0.0560 ± 0.0185	0.0000 ± 0.0000 vs. 0.1083 ± 0.0178	0.0000 ± 0.0000 vs. 0.0868 ± 0.0187	0.0000 ± 0.0000 vs. 0.0000 ± 0.0000	0.0000 ± 0.0000 vs. 0.0000 ± 0.0000
Independent Sample T tests		*t* = −7.198, *p* < 0.05			*t* = −5.237, *p* < 0.05	*t* = −10.541, *p* < 0.01	*t* = -8.033, *p* < 0.01		

G 1: Singleton natural nursing group; G 2: singleton assisted nursing group; G 3: twins assisted nursing group. Data were presented as mean ± S.D. (standard deviation). *n* = 3 per group.
